# Psychometric evaluation of a Theoretical Domains Framework based questionnaire on community pharmacists’ pharmaceutical care service for breastfeeding women

**DOI:** 10.1007/s11096-025-01902-6

**Published:** 2025-03-25

**Authors:** Nazlican Ucar Yaman, Pinar Ay, Mesut Sancar, Derya Büyükkayhan, Betul Okuyan

**Affiliations:** 1 Clinical Pharmacy Department, Institute of Health Sciences, Marmara University, Istanbul, Türkiye; 2 Department of Public Health, School of Medicine, Marmara University, Istanbul, Türkiye; 3 Clinical Pharmacy Department, Faculty of Pharmacy, Marmara University, Istanbul, Türkiye; 4 Division of Neonatology, Department of Pediatrics, Faculty of Medicine, University of Health Sciences, Istanbul, Türkiye

**Keywords:** Behaviour, Breastfeeding, Community pharmacists, Psychometrics, Theoretical domains framework, Validation study

## Abstract

**Background:**

There is a lack of validated Theoretical Domains Framework (TDF) based instruments to investigate barriers and facilitators for community pharmacists (CPs) to provide effective care for breastfeeding women.

**Aim:**

The aim of this study was to evaluate the psychometric properties of a TDF-14 (v2) based questionnaire to identify barriers and facilitators faced by Turkish CPs in provision of pharmaceutical care to breastfeeding women.

**Method:**

This observational study was carried out among CPs in Türkiye. After generating the English form of the questionnaire, translation and cultural adaptation of the questionnaire, an expert panel and pilot study were conducted. Data were collected through an online survey between October 2023 and January 2024. The psychometric properties of the questionnaire were tested by performing test–retest reliability, confirmatory factor analysis (CFA), and internal consistency analysis.

**Results:**

The test–retest reliability analysis (n = 30) indicated that the intraclass correlation coefficient values of each domain were between 0.75 and 0.96 (*p* < 0.001). Four hundred and sixteen CPs completed the questionnaire (response rate: 37.5%). Out of a total of 36 items, six items were excluded. The final questionnaire covered 13 out of the 14 TDF (v2) domains. Chi square/degree of freedom ($\chi$^2^/df), comparative fit index (CFI), root mean square error of approximation (RMSEA), and standardized root mean square residual (SRMR) were 2.01, 0.96, 0.05, and 0.04; respectively. Cronbach's alpha values of each domain ranged from 0.63–0.90.

**Conclusion:**

This TDF-based questionnaire is a valid and reliable tool for evaluating CPs’ enablers and barriers to providing pharmaceutical care to breastfeeding women.

## Impact statements

- This TDF-based questionnaire can be used in identifying enablers and barriers faced by CPs in the provision of pharmaceutical care to breastfeeding women.
- This TDF-based questionnaire can support in developing and tailoring theory-based interventions to address the identified CPs’ barriers in providing pharmaceutical care to breastfeeding women.

## Introduction

Community pharmacists (CPs) play an essential role in the provision of pharmaceutical care to breastfeeding women. This includes conducting medication reviews to identify drug-related problems [1], offering patient education and counselling about medications (including non-prescription medications) [2, 3], and promoting healthy nutrition for both mother and infant [4, 5]. However, CPs face several barriers to providing these services including the lack of knowledge, time, resources, evidence-based information, and concerns related to privacy [2, 6, 7].

The Theoretical Domains Framework (TDF) guides our understanding of the principles of behaviour change which impact implementation. This is what makes it so useful in the development and evaluation of implemented interventions. The TDF is used to investigate the barriers by systematically looking at the determinants of behaviour change and this in turn will guide the most suitable interventions which could be continuing professional development (CPD) programmes or other more suitable interventions [8]. There are two versions of the TDF. While the first version (v1) consists of 12 domains [9] and the second version (v2) includes 14 domains covering 'knowledge', 'skills', 'social/professional role and identity', 'beliefs about capabilities', 'optimism', 'beliefs about consequences', 'reinforcement', 'intentions', 'goals', 'memory, attention and decision processes', 'environmental context and resources', 'social influences', 'emotions', and 'behavioural regulation' [10].

The TDF is among the most used frameworks also in pharmacy practice [11]. This framework has been applied in various pharmacy settings, including immunization provision [12], counselling and management of the homeless population [13], prescribing practices [14], and the adoption of full-scope services [15].

TDF-based questionnaires have previously been developed to identify behavioural determinants of patients and healthcare providers. Huijg et al. (2014) developed items to measure 11 of the 14 TDF (v2) domains [16]. Seward et. al. developed a 61-item questionnaire based on the TDF-14 (v2) to evaluate its use in the implementation of dietary guidelines in childcare settings by using confirmatory factor analysis (CFA) to assess the reliability and validity of the questionnaire [17].

Previously, a Turkish version of the TDF-12 (v1) based questionnaire was developed to identify the CPs’ behavioural determinants in providing pharmaceutical care to older adults [18]. To overcome CPs’ barriers, the results of this study were used to develop a virtual training programme and structured pharmaceutical care service to improve medication adherence, and quality of life in older adults [19]. A TDF-based questionnaire was developed to identify barriers faced by Turkish caregivers in medication administration [20]. Although, a TDF-based framework could be used to investigate the barriers and facilitators of CPs in providing pharmaceutical care to breastfeeding women, there is currently no TDF-14 (v2) based scale in Turkish or other languages to assess the barriers and facilitators that impact their practice.

### Aim

The aim of this study was to evaluate the psychometric properties of a TDF-14 (v2) based questionnaire after translation and cultural adaptation to identify barriers and facilitators faced by Turkish CPs in provision of pharmaceutical care to breastfeeding women. This questionnaire was used to identify the potential barriers and enablers from the perspective of a CP.

### Ethics approval

The study protocol was approved by the Ethical Committee of Marmara University, Institute of Health Sciences (approval date and number: April 19th, 2021- 63). The permission of the Turkish Pharmacists' Association was obtained to conduct this study among the Turkish CPs.

## Method

### Design, setting, study population

This was an observational study that used an online survey to gather data from CPs in Türkiye between October 2023 and January 2024. Informed consent was obtained electronically. An invitation letter was sent to all CPs registered with the Turkish Pharmacists Association and currently working in community pharmacy. The study had no exclusion criteria.

### Data collection

CPs were recruited via LimeSurvey© using the Marmara University Survey System. An online survey was sent via email. The reminder message was sent monthly to closed (private) groups of CPs via social media.

The survey consisted of two sections: the first section gathered participant characteristics (e.g. gender, age, postgraduate education, the duration of working as a CP and the frequency of pharmacy visits by breastfeeding women) and the second section contained a TDF-based questionnaire. At the beginning of the survey, the definition of pharmaceutical care and the list of roles of CPs in pharmaceutical care services to breastfeeding women were presented.

The English form of the TDF-based questionnaire was developed on the basis of existing valid and reliable instruments [16, 18, 21]. The questionnaire had 36 items based on the 14-domain TDF (v2). There were either two or three items per domain relating to enablers and barriers of CPs in providing pharmaceutical care to breastfeeding women. Each item was rated on a 7-point Likert scale ranging from 1 (strongly disagree) to 7 (strongly agree). The Turkish translation and cultural adaptation of the questionnaire was conducted by following the guidelines of the International Society for Pharmacoeconomics, and Outcomes Research (ISPOR) Task Force [22]. The two native Turkish speakers who were fluent in English, translated the TDF-based questionnaire into Turkish. The back translation into English was carried out by two native English speakers who were fluent in Turkish. The understandability of the questionnaire was evaluated by an expert group (n = 5; consisting of clinical pharmacists and a paediatrician). A pilot study was conducted with 20 pharmacists to test the understandability of the items (using criteria such as relevance, clarity, acceptability, and ease of use). Some minor grammatical changes were made. These pharmacists were excluded from either the test–retest or the psychometric study. The TDF-based questionnaire was evaluated for test–retest reliability over two-week period in 30 CPs (not included in the psychometric study). The questionnaire took approximately 10–15 min to complete.

### Sample size calculation

Streiner et al. (2015) recommend a sample size of 10 tenfold the total number of items [23]. As the questionnaire contained 36 items, the minimum sample size was calculated to be 360 CPs.

### Data analysis

Data were analysed using SPSS 29.0 (free trial) and jamovi version 2.4.14.0 open access statistical software (https://www.jamovi.org). Descriptive statistics were presented as frequencies and percentages. Continuous variables were shown as medians (interquartile range [IQR]). Each domain of the TDF-based questionnaire was identified as a barrier if the median value for that domain was less than 4 out of 7 (1 = strongly disagree; 4 = neither disagree nor agree; 7 = strongly agree) [24]. The first three options refer to the level of disagreement, which was identified as a barrier.

Test–retest reliability was determined by using the Wilcoxon test and the intraclass correlation coefficient (ICC [95% confidence interval, CI]). Statistical significance was evaluated on the basis of *p* ≤ 0.05. The construct validity of the questionnaire was conducted using confirmatory factor analysis (CFA). Items were removed from the questionnaire if the factor loading was less than 0.4 and there was no significant loading (*p* > 0.05) [23]. Goodness of fit was evaluated by using Chi square/degree of freedom ($\chi$^2^/df), standardized root mean square residual (SRMR), root mean square error of approximation (RMSEA), and comparative fit index (CFI). Internal consistency was assessed by measuring Cronbach's alpha values (≥ 0.7 was considered acceptable) [25].

## Results

ICC values for each domain in the test–retest reliability analysis (n = 30) were between 0.75 and 0.96 (*p* < 0.001). There was no statistically significant difference between the test–retest measures (*p* > 0.05). The ICC coefficients of the domains of the questionnaire according to test–retest reliability are presented in Table 1.

**Table 1:** ICC coefficients of the domains in the questionnaire according to test–retest reliability (n = 30)

TD-14 (v2) domains Items	ICC (95% CI)
Knowledge	0.86 (0.71–0.93)^a,b^
Skills	0.89 (0.76–0.95)^a,b^
Social/Professional role and identity	0.80 (0.58–0.91)^a,b^
Beliefs about capabilities	0.90 (0.80–0.95)^a,b^
Beliefs about consequences	0.84 (0.66–0.92)^a,b^
Intentions	0.78 (0.54–0.89)^a,b^
Goals	0.89 (0.77–0.95)^a,b^
Memory, attention and decision processes	0.87 (0.72–0.94)^a,b^
Environmental context and resources	0.75 (0.49–0.88)^a,b^
Social influences	0.85 (0.69–0.93)^a,b^
Emotions	0.88 (0.75–0.94)^a,b^
Behavioural regulation	0.92 (0.83–0.96)^a,b^
Optimism	0.84 (0.66–0.92)^a,b^

^a^*p* < 0.001; ^b^Wilcoxon signed rank test used *p* > 0.05; ICC: Intraclass  Correlation; CI: Confidence interval

One thousand one hundred and eight participants were invited to the study and 416 completed the TDF-based questionnaire (response rate: 37.5%). The majority were female (n = 290; 69.7%) and had 5 years or less experience as a CP (n = 248; 59.6%). More than half of the participants reported that breastfeeding women always/often visited their pharmacy (n = 226; 54.3%). The characteristics of the CPs are presented in Table 2.

**Table 2:** Characteristics of the CPs (n = 416)

	n (%)
*Gender*	
Female	290 (69.7)
Male	126 (30.3)
Age (years), Median [IQR]	31.0 [27.0–39.0]
*Postgraduate education*	
MSc	55 (13.2)
PhD	10 (2.4)
*Experience as a CP*	
≤ 5 years	248 (59.6)
6–20 years	113 (27.2)
> 20 years	55 (13.2)
*How often breastfeeding women visit their pharmacy*	
Always/often	226 (54.3)
Sometimes	130 (31.3)
Never/seldom	60 (14.4)

IQR: interquartile range

The median values for each domain ranged from 6.50 [6.00–7.00] to 2.00 [1.00–4.00]. The environmental context and resources domain was a barrier based on its median value (2.00 [1.00–4.00]). Chi-square/degree of freedom ($\chi$^2^/df) was 2.01 (acceptable fit: 2 ≤ $\chi$^2^/df ≤ 3; *p* < 0.001). CFI was 0.96 (goodness-of-fit: 0.95 ≤ CFI ≤ 1.00). RMSEA was 0.05 (goodness-of-fit: 0.00 ≤ RMSEA ≤ 0.05). The SRMR was 0.04 (goodness-of-fit: 0.0 ≤ SRMR ≤ 0.0.5). Out of a total of 36 items, six items (reinforcements [n = 2], emotions [n = 3], goals [n = 1]) were excluded because the factor loadings were less than 0.4. Cronbach's alpha values for each domain ranged from 0.63 to 0.90. The descriptive analysis, factor loadings and Cronbach’s alpha coefficients of the items in the TDF-based questionnaire are presented in Table 3.

**Table 3:** The descriptive analysis, factor loadings and Cronbach’s alpha coefficients of the items in the TDF-based questionnaire (n = 416)

TDFv2 domains items	Factor loading	Cronbach’s alpha	Median [IQR]
*Knowledge*		**0.89**	5.33 [5.00–6.00]
I am aware of the content and objectives of pharmaceutical care delivered to breastfeeding women	0.87	0.84*	6.00 [5.00–6.00]
I know the content and objectives of pharmaceutical care delivered to breastfeeding women	0.87	0.84*	6.00 [5.00–6.00]
I know how to deliver pharmaceutical care to breastfeeding women during visits to the community pharmacy	0.84	0.87*	5.00 [5.00–6.00]
*Skills*		**0.82**	5.00 [3.00–5.50]
I have been trained based on case scenarios of how to deliver pharmaceutical care to breastfeeding women during visits to the community pharmacy	0.87	–*	4.00 [2.00–6.00]
I have been trained how to deliver pharmaceutical care to breastfeeding women during visits to the community pharmacy	0.80	–*	5.00 [3.25–6.00]
*Social/Professional role and identity*		**0.90**	6.33 [5.66–7.00]
As a pharmacist, it is my job to deliver pharmaceutical care to breastfeeding women during visits to the community pharmacy	0.92	0.81*	7.00 [6.00–7.00]
Delivering pharmaceutical care to breastfeeding women during visits to the community pharmacy is part of my work as a pharmacist	0.91	0.83*	6.00 [6.00–7.00]
My role in delivering pharmaceutical care to breastfeeding women in the community pharmacy is clear to me	0.77	0.91*	6.00 [5.00–7.00]
*Beliefs about capabilities*		**0.83**	5.00 [4.33–6.00]
I am confident that if I wanted, I could deliver pharmaceutical care to breastfeeding women during visits to the community pharmacy in my current workflow	0.84	0.72*	5.00 [5.00–6.00]
I am confident that I can deliver pharmaceutical care to breastfeeding women during visits to the community pharmacy even when there is little time	0.82	0.83*	5.00 [4.00–6.00]
I am confident that I can deliver pharmaceutical care to breastfeeding women during visits to the community pharmacy even when they are not motivated	0.72	0.76*	5.00 [4.00–6.00]
*Optimism*		**0.65**	6.00 [5.00–6.50]
With regard to delivering pharmaceutical care to breastfeeding women during visits to the community pharmacy, I usually expect the best	0.82	–*	6.00 [5.00–7.00]
With regard to delivering pharmaceutical care to breastfeeding women during visits to the community pharmacy, I’m always optimistic about the future	0.59	–*	6.00 [5.00–7.00]
*Beliefs about consequences*		**0.73**	6.50 [6.00–7.00]
If I deliver pharmaceutical care to breastfeeding women during visits to the community pharmacy, it will benefit public health	0.83	–*	7.00 [6.00–7.00]
If I deliver pharmaceutical care to breastfeeding women during visits to the community pharmacy, they will appreciate this	0.69	–*	6.00 [6.00–7.00]
*Intentions*		**0.90**	6.00 [5.33–6.66]
I intend to deliver pharmaceutical care to breastfeeding women in their next visit to the community pharmacy	0.89	0.85*	6.00 [6.00–7.00]
I will definitely deliver pharmaceutical care to breastfeeding women in their next to visit the community pharmacy	0.88	0.88*	6.00 [5.00–7.00]
I intend to deliver pharmaceutical care in the community pharmacy to the next 10 breastfeeding women	0.84	0.86*	6.00 [5.00–7.00]
*Goals*		**0.89**	5.00 [4.00–5.50]
I have a clear plan for which breastfeeding women who visit the community pharmacy, I will provide pharmaceutical care	0.91	–*	5.00 [4.00–6.00]
I have a clear plan when I will deliver pharmaceutical care to breastfeeding women during their visits to the community pharmacy	0.88	–*	5.00 [4.00–6.00]
*Memory, attention and decision processes*		**0.78**	5.50 [4.00–6.00]
I often forget to deliver pharmaceutical care to breastfeeding women in the community pharmacy**	0.84	–*	5.00 [4.00–6.00]
When a breastfeeding woman visits the pharmacy, I have difficulty blocking attention to providing her with pharmaceutical care**	0.77	–*	6.00 [4.00–6.00]
*Environmental context and resources*		**0.85**	2.00 [1.00–4.00]
There is sufficient financial support (e.g., chamber of pharmacists, health authorities, insurance companies) for pharmaceutical care to a breastfeeding woman as part of accepted pharmacy practice	0.89	–*	2.00 [1.00–4.00]
Within the socio-political context, there is sufficient financial support (e.g., chamber of pharmacists, health authorities, insurance companies) for information resources	0.84	–*	2.00 [1.00–4.00]
*Social influences*		**0.63**	5.50 [4.50–6.00]
Most people whose opinion I value would approve me of delivering pharmaceutical care to breastfeeding women when they visit the community pharmacy	0.87	–*	6.00 [5.00–6.00]
Most people who are important to me think that I should deliver pharmaceutical care to breastfeeding women during their visits to the community pharmacy	0.55	–*	5.00 [4.00–6.00]
*Emotions*		**0.82**	4.00 [2.50–5.00]
When I am delivering pharmaceutical care to breastfeeding women, I feel agitated about receiving negative feedback from breastfeeding women**	0.88	–*	3.00 [2.00–6.00]
When I am delivering pharmaceutical care to breastfeeding women, I feel agitated about receiving negative feedback from doctors**	0.79	–*	4.00 [2.00–6.00]
*Behavioural regulation*		**0.70**	5.00 [4.00–6.00]
I keep track of my professional progress with regard to delivering pharmaceutical care to breastfeeding women in the community pharmacy	0.79	–*	5.00 [5.00–6.00]
I check regularly whether I am getting closer to delivering pharmaceutical care to breastfeeding women in the community pharmacy	0.70	–*	5.00 [4.00–6.00]

*Cronbach’s alpha if item deleted; ** Reversed items; IQR: interquartile range

The Cronbach alpha values for each domain were written in bold

## Discussion

This TDF-based questionnaire is a valid and reliable tool for assessing CPs' facilitators and barriers in providing pharmaceutical care to breastfeeding women. The final questionnaire covered 13 of the 14 TDF (v2) domains.

The questionnaire was developed based on the version 2 of the TDF. Although the sample size was adequate, the response rate was relatively low. This may affect the generalisability of the findings. The study population consisted mainly of younger CPs with less experience compared to another study conducted in Türkiye [18]. CPs with more experience may lack the necessary knowledge and skills to provide pharmaceutical care, as pharmaceutical care courses have only been made mandatory in bachelor programmes in Türkiye in the last decade. In the previous study [18], the knowledge and skills of CPs to provide pharmaceutical care was found to be a barrier, in contrast to the present study. The barriers faced by older CPs in providing pharmaceutical care may be underrepresented in this study. Therefore, we need to test this questionnaire in populations that include older CPs. The online self-reported questionnaire could lead to social desirability bias and recall bias and CPs with low digital literacy may not respond to the survey. The present tool was generated by using the TDF-based questionnaires developed in previous studies. However, mixed methods or qualitative studies are needed to identify additional barriers related to the provision of pharmaceutical care to breastfeeding women as many of the barriers and enablers are likely to be cultural [26–28].

The Cronbach's alpha values for the optimism and social influence domains were lower than 0.7. Streiner et al. suggested that lower values of Cronbach’s alpha values are acceptable in domains with few items [23, 29]. These domains had two items as suggested by Huijg et al. [16]. Although the number of items in the study by Seward et al. [17] was higher than in the present study, the Cronbach's alpha values for these two domains were consistent with the present study. Isenor et al. [14] did not calculate Cronbach’s alpha values for domains of the TDF-based questionnaire if the domain had less than three items. Hussein et al. [15] also found that the Cronbach’s alpha values of five domains in a TDF-based questionnaire, consisting of 2–3 items, were less than 0.7.

All the items for the reinforcement domain were excluded from the questionnaire due to their low factor loadings. Therefore, the final questionnaire covered 13 out of the 14 TDF (v2) domains. This may be due to the professional role of CPs in Türkiye where they are primarily responsible for dispensing prescriptions and do not receive recognition or financial reimbursement for pharmaceutical care services. The item related to this domain was determined as a barrier also in a previous study conducted in Türkiye [18]. All ICC values were acceptable (> 0.7). All CFA model fit indices of the questionnaire in the present study were satisfactory in contrast to the questionnaire in the previous study [17].

The environmental context and resources domain was found to be a barrier for CPs in the provision of pharmaceutical care to breastfeeding women. This finding is also consistent with a previous study [18]. In order to provide this service, CPs needed sufficient funding from the Turkish Pharmacists Association, the Ministry of Health, and the reimbursement institutions. According to the Theory & Techniques Tool [30], social support (practical), restructuring the physical environment, restructuring the social environment, and adding objects to the environment could be selected as appropriate behaviour change techniques (BCTs) [31]. In well-designed cross-sectional studies, this TDF-based questionnaire can be used to develop and tailor theory-based interventions to address the identified CPs’ barriers in providing pharmaceutical care to breastfeeding women [32].

## Conclusion

This TDF-based questionnaire is a valid and reliable tool for assessing CPs' facilitators and barriers in providing pharmaceutical care to breastfeeding women. The final questionnaire covered 13 of the 14 TDF (v2) domains. Using this questionnaire, theory-based interventions could be developed to address barriers and support CPs in the provision of pharmaceutical care to breastfeeding women.
